# Asthma management among allergists in Italy: results from a survey

**DOI:** 10.1186/s12948-017-0067-2

**Published:** 2017-05-08

**Authors:** M. S. Magnoni, M. Caminati, G. W. Canonica, F. Arpinelli, A. Rizzi, G. Senna

**Affiliations:** 1 Medical and Scientific Department, GlaxoSmithKline, Verona, Italy; 2 Asthma Center and Allergy Unit, Verona University Hospital, Verona, Italy; 3 Personalized Medicine Asthma & Allergy Clinic, Humanitas University-Research Hospital, Rozzano, Milano, Italy

**Keywords:** Asthma, Online survey, Follow-up and monitoring tools, Treatment, Adherence

## Abstract

**Background:**

In Europe more than 50% of asthmatic treated patients have not well-controlled asthma. Asthma affects about 2.5 million of patients in Italy.

**Aims and objectives:**

The present survey aims at investigating how Italian allergists approach asthmatic patients, in order to highlight pitfalls and unmet needs concerning real-life asthma management.

**Methods:**

An anonymous 16 item web questionnaire was available (April–October 2015) to all allergists who visited the web site of SIAAIC (Società Italiana di Allergologia, Asma Immunologia Clinica). Those who wished to give their contribution had the opportunity to answer about epidemiology, risk factors, treatment approaches, and adherence to therapy.

**Results:**

One hundred and seventy four allergists answered the survey. 54% of them reported up to 10 patient visits per week and 35.3% between 10 and 30. The most frequent reasons of follow up visits are routine check-up (56.5% of allergists), and worsening of symptoms (41% of allergists). Nocturnal apnoeas, gastro-esophageal reflux and obesity are the most important comorbidities/risk factors of poorly controlled asthma. Bronchial hyper-responsiveness, increased NO levels and reduced exercise tolerance are the most important indicators of asthma severity. Concerning therapy, ICS combined with LABA is the treatment of choice suitable for the majority of patients. A rapid onset of action and a flexible ICS dosage are indicated as the optimal characteristics for achieving the therapeutic goals. Poor adherence to therapy is an important reason for symptom worsening for the majority of allergists. Complex dosage regimens and economic aspects are considered the most important factors impacting on adherence.

**Conclusions:**

Allergists are involved in the management of asthma, regularly seeing their patients. Co-morbidities are frequent in asthmatic patients and may impact negatively on disease control, thus identifying patients who need a more careful and strict monitoring. Airway hyper-responsiveness to methacholine challenge test and nitric oxide are considered important indicators of asthma severity. The combination of LABA and inhaled steroids is considered the treatment of choice for most asthmatic patients, in keeping with broad evidence indicating that the combination therapy is more effective and rapid in gaining asthma control than inhaled corticosteroids alone. Adherence to medication regimens is considered of essence to achieve the therapeutic goals.

**Electronic supplementary material:**

The online version of this article (doi:10.1186/s12948-017-0067-2) contains supplementary material, which is available to authorized users.

## Background

Asthma is one of the most prevalent chronic conditions, affecting approximately 300 million people worldwide [1]. In developed countries its prevalence is estimated to be between 4 and 12% in adults and 10–15% in children [2]. In Italy the overall estimate is 5% [3], corresponding to around 2.5 million patients.

Asthma cannot be cured, but appropriate management can control the disorder and enable people to enjoy a good quality of life. However, despite the advances in therapeutic approaches and the dissemination of guidelines [4], observational studies have shown that in Europe more than 50% of treated patients report not well-controlled asthma [5], causing substantial limitations in daily life, in terms of reduced productivity at work or school and frequent inability to perform normal activities.

The cornerstone of asthma management is to achieve and maintain the control of the disease [4] and several specialists, including allergists, should contribute to this goal. The present survey aims at investigating how Italian allergists approach patients with asthma, in order to point out pitfalls and unmet needs concerning real-life management of the disease.

## Methods

A board of experts belonging to Società Italiana di Allergologia, Asma Immunologia Clinica (SIAAIC) developed a questionnaire composed of 16 multiple choice questions covering epidemiological (number of patients assisted) and clinical (presence of co-morbidities or risk factors) aspects about asthma, and exploring the overall management strategies (follow up, treatments, adherence) adopted by the Italian allergists (see Additional file 1: Appendix S1).

All the SIAAIC members were invited to complete the survey through an email invitation letter. Between April and October 2015 the questionnaire was available to all allergists who visited the web site of SIAAIC. The questionnaire was anonymous. All the data and answers entered into the system were checked for consistency and completeness and each respondent could only complete the questionnaire once.

In this paper we report a descriptive analysis of the data.

## Results

A total of 174 questionnaires have been analyzed; this sample represents 25% of SIAAIC Allergists (SIAAIC is the scientific society that collects the highest number of Italian allergists).

### Number of visits

**Fig. 1:**
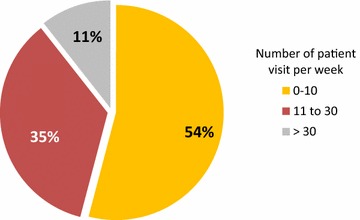
Percentage of allergists according to the number of patient visits per week

**Fig. 2:**
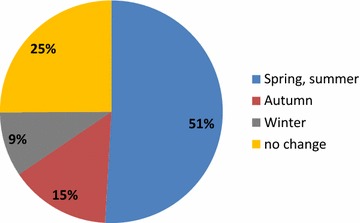
Percentage of allergists reporting the period of the year with increased number of patients with respiratory symptoms

Ninety allergists (54%) reported to see up to 10 asthma patients per week and 60 (35.3%) between 10 and 30 patients (Fig. 1). During the pollen season, the number of visits increased for 87 allergists (51%), whereas for 43 (25%) it remained constant over the year (Fig. 2).

### Follow-up and risk factors for poorly controlled asthma

The most frequent reasons of follow up visits were routine checkup for 91 allergists (56.5%), and worsening of symptoms for 66 allergists (41%), whereas the percentage of visits due to drug adverse effects was negligible (3%).

Around 70% of allergists declared to assess their patients regularly with scheduled follow up visits: 25–50% of patients according to 54 allergists (35.5%) and >50–75% of patients according to 50 allergists (33.8%).

**Fig. 3:**
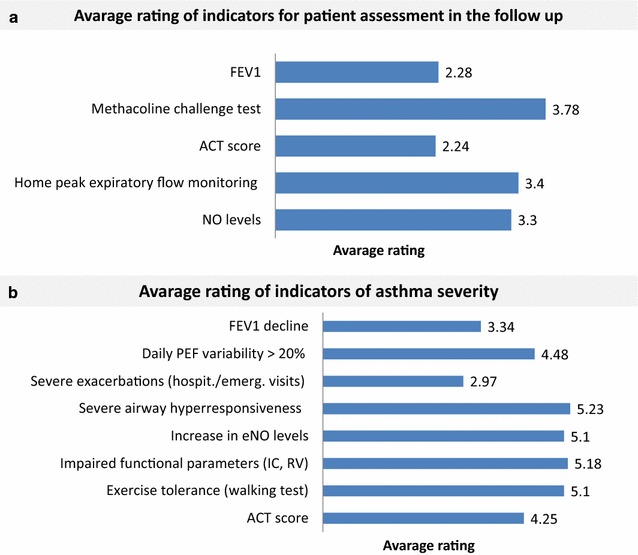
Avarage rating of indicators (in order of importance) for the assessment of asthmatic patients during the follow up (**a**) and for staging of asthma severity (**b**)

For the assessment of patient’s condition in the follow up, airway hyper-responsiveness by methacholine challenge testing was considered the most important indicator, followed by nitric oxide (NO) levels and home PEF monitoring (Fig. 3a). Accordingly, airway hyper-responsiveness and NO levels were also rated as the most important indicators of asthma severity, together with exercise tolerance and lung volumes (residual volume, inspiratory capacity), whereas severe exacerbations had the lowest rate (Fig. 3b).

**Fig. 4:**
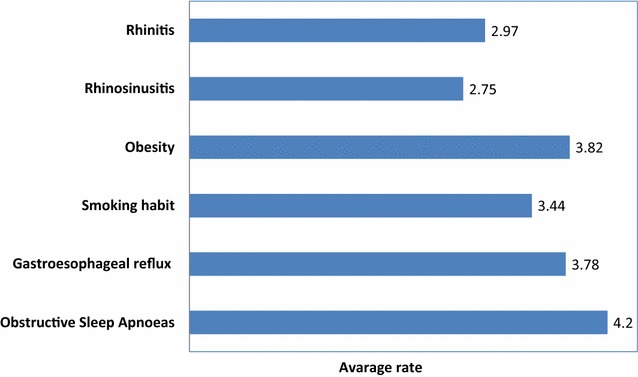
Avarage rating of co-morbidities/risk factors impacting on asthma control (in order of importance)

Nocturnal apneas and obesity were rated as the most important co-morbidities/risk factors of poorly controlled asthma, followed by gastro-esophageal reflux and smoking habits and lastly by rhinitis and rhino-sinusitis (Fig. 4).

### Treatment and adherence

**Fig. 5:**
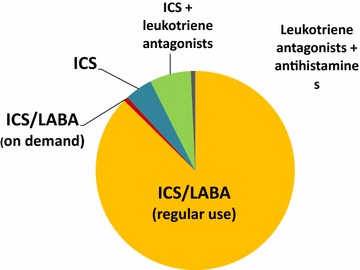
Asthma treatment according to Italian allergists (% of allergists)

**Fig. 6:**
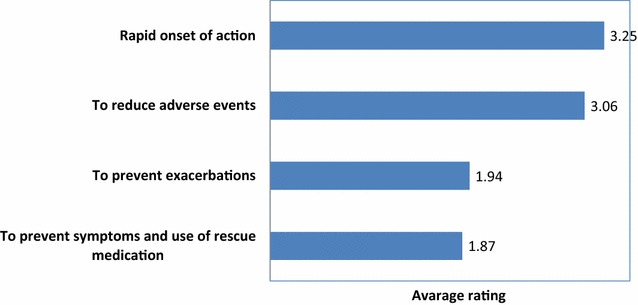
Main therapeutic goals for the asthmatic patient (in order of importance)

**Fig. 7:**
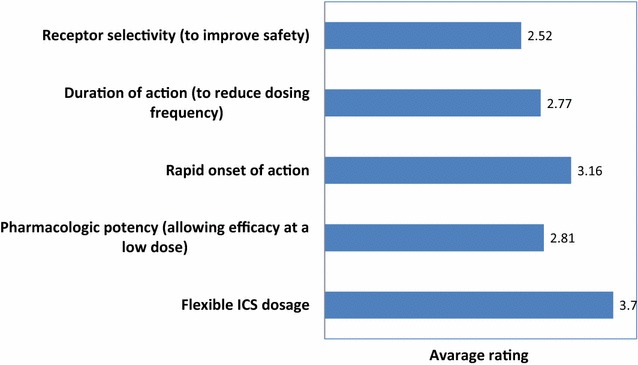
Optimal drug features to achieve therapeutic goals (in order of importance)

Concerning therapy, regular use of inhaled corticosteroids (ICS) combined with long acting beta agonists (LABA) was considered the treatment of choice suitable for the majority of patients (Fig. 5). A rapid onset of action and a good safety profile were rated as most important therapeutic goals than the prevention of exacerbations and rescue medication use (Fig. 6). A flexible ICS dosage and a rapid onset of action were indicated as optimal characteristics for achieving the therapeutic goals (Fig. 7).

**Fig. 8:**
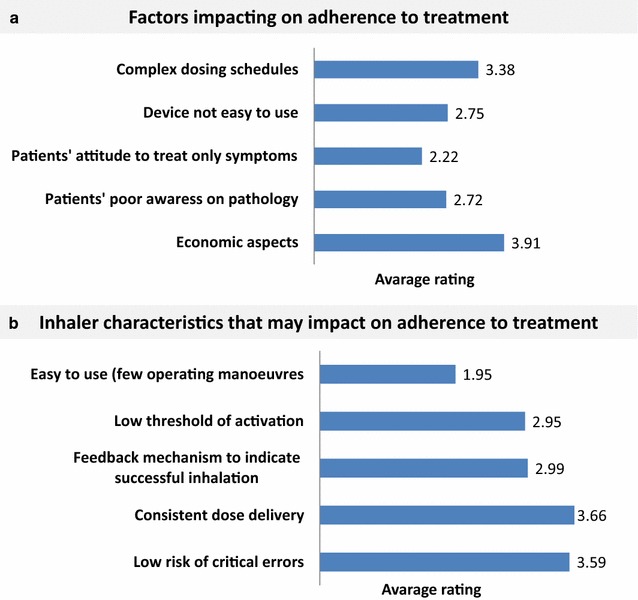
Factors impacting on adherence (**a**) and inhaler characteristics that may impact on adherence (**b**)

Poor adherence to therapy was considered an important cause of symptom worsening for the majority of allergists, and 37% of them (n. 56) believed that it was the prevalent cause. Notably, complex dosing regimens and cost of therapy were rated as the most important factors impacting on adherence (Fig. 8a).

As regards the inhaler, consistency of the released dose and low number of critical errors were considered the most important characteristics impacting on adherence (Fig. 8b).

## Discussion

Allergists are involved in the management of asthma, seeing their patients more frequently, but not exclusively, in the pollen season. Interestingly, they rely on airway hyper-responsiveness and inflammation for classification of asthma severity and monitoring of asthma control during follow-up visits, as indicated by the high rate attributed to methacholine challenge testing and NO levels. Indeed, the degree of airway hyper-responsiveness and inflammation are usually in proportion to the severity of the underlying asthma and may reflect the response to the treatment [6]. Of note, also changes in lung volumes, indicators of pulmonary hyperinflation, which is responsible for low tolerance to physical efforts, are rated as important markers of asthma severity, whereas severe exacerbations seem to be less relevant. Allergists mostly manage mild to moderate allergic asthma and their patients are regularly followed up in order to prevent exacerbations triggered by allergen exposure. Thus, in comparison with respiratory medicine specialists who usually deal with severe non-allergic refractory asthma, allergists are probably less involved in the management of severe exacerbations. This may account for the low rate attributed to asthma exacerbations as a marker of control.

Consistently with international reports [7–10], allergists are aware that risk factors and co-morbidities may impact on the natural history of asthma, leading to symptoms worsening and reduced treatment efficacy, thus identifying patients who need a more careful and strict monitoring to maintain control. It is intriguing that rhinitis and smoking habits are considered less impactful on asthma control than nocturnal apneas and obesity: possibly, the latters are regarded as risk factors/comorbidities which are more difficult to control or cure.

For allergists the combination of LABA and ICS is the treatment of choice for most asthmatic patients. This is consistent with broad literature evidence indicating that that combination therapy is the most effective strategy in preventing clinical manifestations and gaining rapid asthma control than ICS alone [11]. Furthermore, a flexible ICS dosage is considered a key point in the management of asthma, allowing titration according to the level of clinical manifestation and airway inflammation, which is the dominant process in asthma. On the other hand, a high percentage of allergists rate a rapid onset of action as a more important therapeutic goal than the reduction of exacerbations and the prevention of symptoms and use of rescue medication, which are sensitive markers of poorly controlled asthma according to international guidelines. This was an unexpected finding. Clearly, a rapid onset of action is essential in the management of acute asthma but less critical for chronic therapy. We speculate that this finding could be attributed to the fact that allergists generally manage mild to moderate allergic asthma, mostly characterized by seasonal symptoms, and therapeutic strategies are often tailored accordingly. Under this perspective, a rapid onset of action might be considered as an added value of the treatment.

Like in every chronic disease, adherence to medication regimens is considered of essence to achieve and maintain asthma control [12]. Indeed, there is no doubt that poor adherence to medication regimens represents a significant barrier to optimal management of chronic respiratory diseases, contributing to a substantial worsening of symptoms, reduction in health-related quality of life and increase in healthcare costs [12, 13]. Complex dosing regimens have been rated are rated as major factors impacting on adherence to medication, as suggested by several studies [14, 15]. Indeed, multiple-inhaler use has been associated with higher rates of non-adherence than single-inhaler use in both asthma and COPD patients, potentially because of the increased complexity introduced by the additional inhaler(s). Also medication dosing frequency has important effects on adherence: the simplification of medication dosing seems to be the single intervention with the strongest impact on adherence, with once-daily regimes resulting in up to twice as many adherent days as more frequent dosing regimens [16]. Interestingly, also economic aspects have been considered important for adherence, possibly with reference to intranasal steroids for the treatment of comorbid rhinitis. Since only a small proportion of follow up visits was due to drug related side effects, they do not seem to be one of the drivers for poor adherence.

In regards to inhaler preferred features, allergists give high importance to inhaler efficiency in terms of consistency in dose delivery across different flow rates, making the device suitable and reliable also in patients with severe airflow limitation. Another important feature is the low risk of critical errors that impair the delivery of medication to the lung.

Our survey has some limitations: (1) it has been addressed only to allergists, and we are aware that it would be of interest to compare the responses of different kinds of specialists (pneumologists, general practitioners) on the management of asthma; (2) the questionnaire did not investigate the knowledge of severe asthma, thus information is missing about the management of this kind of patients by allergists; (3) like in all surveys, the responses from physicians may not always correspond to their daily clinical practice, but rather reflect their opinions and insights on optimal disease management.

## Conclusions

Allergists are involved in the management of asthma, regularly seeing their patients. Co-morbidities may identify patients who need a more careful and strict monitoring to achieve asthma control. The combination of LABA and inhaled steroids is considered the treatment of choice for most asthmatic patients, in keeping with broad literature evidence indicating that the combination therapy is more effective and rapid in gaining asthma control than inhaled corticosteroids alone. Adherence to medication regimens is considered of essence to achieve the therapeutic goals. Complex dosing regimens and poor inhalation technique may negatively impact on adherence.

## Additional file

**Additional file 1: Appendix S1.** Questionnaire.

## Abbreviations

ICS: inhaled corticosteroids
LABA: long acting beta agonists
NO: nitric oxide
SIAAIC: Società Italiana di Allergologia, Asma Immunologia Clinica
