# Supplementary material for: Asthma management among allergists in Italy: results from a survey
Source: Clin Mol Allergy. 2017 May 8;15:11. doi: 10.1186/s12948-017-0067-2 (PMC5422900; doi:10.1186/s12948-017-0067-2)
Supplement: Supplementary file 1 — Additional file 1: Appendix S1. Questionnaire. [file 12948_2017_67_MOESM1_ESM.docx]

**Appendix**

1. **How many patients with symptoms consistent with bronchial asthma (cough, dyspnea, wheezing) do you see in you clinic on average in a week?**

€0-10 €10-30 €> 30

1. **The patients coming to your surgery/clinic, mainly come from:**

- Emergency Room
- Other Specialists
- Not specified

1. **At what time of the year, compared to the normal trend, do respiratory symptoms increase in your patients with asthma?**

- Spring, summer
- No significant change
- Autumn
- Winter

1. **For asthmatic patients, what is the most frequent cause of subsequent visits to the first one?**

- Exacerbations/worsening of symptoms
- Routine check
- Side effects related to prescribed therapy

1. **How the following co-morbidities/risk factors may adversely affect the control of asthma in your patients (in order of importance)**

- Rhinitis
- Rhinosinusitis
- Obesity
- Smoking habit
- Gastroesophageal reflux
- Obstructive sleep apnoeas

1. **In the event that the second visit is due to the worsening of symptoms or exacerbations, in what proportion of patients poor adherence to treatment was the main cause?**

- 81 to 100%
- 61% to 80
- 41% to 60%
- 21% to 41%
- < 20%

1. **In your experience, poor adherence to therapy is mainly due to which of the following factors (in order of importance)**:

- Complex dosing schedule
- Device not easy to use
- Patients’ attitude to treat only symptoms
- Patients’ poor awareness on pathology
- Economica aspects

1. **What features should an ideal inhaler have to favor therapeutic adherence? (in order of importance)**

- Easy to use (few operating manoeuvres )
- Low threshold of activation
- Feed-back mechanism to indicate successful inhalation
- Consistent dose delivery
- Low risk of critical errors

1. **In your experience, what proportion of patients has a regular follow up**?

- <10%
- 10% to 25%
- 26% to 50%
- 51% to 75%
- 76% to 100%

1. **In your clinical practice, how do you mainly schedule the follow up visits**?

- Verbal recommendation of a follow-up visit
- Scheduling the next appointment
- Availability of email address / phone number for the visit program
- Facebook page/personal website dedicated to communication with patients
- Other (speciy)

1. **What indicators do you consider most relevant in the assessment of the patients during the follow-up? (in order of importance)**

- FEV1
- Methacoline challenge test
- ACT score
- Home peak expiratory flow monitoring
- NO levels

1. **In your clinical practice, which of the following tools do you mainly use to inform the patients about the risk factors?**

- Verbal information
- Brochure
- Structured educational meetings
- Connection to certified websites
- Other (specify)

1. **In your opinion, what are the most reliable indicators of severity of asthma? (in order of importance)**

- FEV1 decline
- Daily PEF variability > 20%
- Severe exacerbations (hospitalizations/emergency visits)
- Severe airway hyperresponsiveness
- Increase in eNO levels
- Impaired functional parameters (IC, RV)
- Exercise tolerance (walking test)
- ACT score < 20

1. **In the treatment of patients with asthma, what are the main goals? (in order of importance)**

- To prevent symptoms and use of rescue medication
- To prevent exacerbations
- To reduce adverse events of medications
- Rapid onset of action of the medication

1. **What are the features of an optimal treatment for the achievement of the therapeutic goals (in order of importance):**

- Receptor selectivity (to improve safety)
- Duration of action (to reduce dosing frequency)
- Rapid onset of action
- Pharmacologic affinity/potency (allowing efficacy at a low dose)
- Flexible I CS dosage

1. **What is the pharmacological approach that can be effective in most of your patients with asthma?**

- Regular treatment with ICS/LABA fixed combination
- ICS/LABA fixed combination used on demand
- Inhaled corticosteroids (ICS)
- ICS + leukotriene antagonists
- Leukotriene antagonists +antihistamines
